# Absorption, Metabolism, and Excretion of ACT-1004-1239, a First-In-Class CXCR7 Antagonist: *In Vitro*, Preclinical, and Clinical Data

**DOI:** 10.3389/fphar.2022.812065

**Published:** 2022-03-30

**Authors:** Christine Huynh, Swen Seeland, Jerome Segrestaa, Carmela Gnerre, Jens Hogeback, Henriette E. Meyer zu Schwabedissen, Jasper Dingemanse, Patricia N. Sidharta

**Affiliations:** ^1^ Department of Clinical Pharmacology, Idorsia Pharmaceuticals Ltd., Allschwil, Switzerland; ^2^ Department of Pharmaceutical Sciences, Biopharmacy, University of Basel, Basel, Switzerland; ^3^ Department of Preclinical Drug Metabolism and Pharmacokinetics, Idorsia Pharmaceuticals Ltd., Allschwil, Switzerland; ^4^ A&M Labor für Analytik und Metabolismusforschung Service GmbH, Bergheim, Germany

**Keywords:** CXCR7, ADME, accelerator mass spectrometry, microtracer, CYP3A4, ^14^C-ACT-1004-1239, first-in-human

## Abstract

ACT-1004-1239 is a potent, selective, first-in-class CXCR7 antagonist, which shows a favorable preclinical and clinical profile. Here we report the metabolites and the metabolic pathways of ACT-1004-1239 identified using results from *in vitro* and *in vivo* studies. Two complementary *in vitro* studies (incubation with human liver microsomes in the absence/presence of cytochrome P450- [CYP] specific chemical inhibitors and incubation with recombinant CYPs) were conducted to identify CYPs involved in ACT-1004-1239 metabolism. For the *in vivo* investigations, a microtracer approach was integrated in the first-in-human study to assess mass balance and absorption, distribution, metabolism, and excretion (ADME) characteristics of ACT-1004-1239. Six healthy male subjects received orally 100 mg non-radioactive ACT-1004-1239 together with 1 μCi ^14^C-ACT-1004-1239. Plasma, urine, and feces samples were collected up to 240 h post-dose and ^14^C-drug-related material was measured with accelerator mass spectrometry. This technique was also used to construct radiochromatograms of pooled human samples. Metabolite structure elucidation of human-relevant metabolites was performed using high performance liquid chromatography coupled with high resolution mass spectrometry and facilitated by the use of rat samples. CYP3A4 was identified as the major CYP catalyzing the formation of M1 *in vitro*. In humans, the cumulative recovery from urine and feces was 84.1% of the dose with the majority being eliminated via the feces (69.6%) and the rest via the urine (14.5%). In human plasma, two major circulating metabolites were identified, i.e., M1 and M23. Elimination *via* M1 was the only elimination pathway that contributed to ≥25% of ACT-1004-1239 elimination. M1 was identified as a secondary amine metabolite following oxidative N-dealkylation of the parent. M23 was identified as a difluorophenyl isoxazole carboxylic acid metabolite following central amide bond hydrolysis of the parent. Other metabolites observed in humans were A1, A2, and A3. Metabolite A1 was identified as an analog of M1 after oxidative defluorination, whereas both, A2 and A3, were identified as a reduced analog of M1 and parent, respectively, after addition of two hydrogen atoms at the isoxazole ring. In conclusion, CYP3A4 contributes to a relevant extent to ACT-1004-1239 disposition and two major circulating metabolites were observed in humans.

**Clinical Trial Registration:** (https://clinicaltrials.gov/ct2/show/NCT03869320) ClinicalTrials.gov Identifier NCT03869320.

## Introduction

ACT-1004-1239 is an orally available, potent, selective, insurmountable, small-molecule CXCR7 antagonist (Richard-Bildstein et al., 2020). Preclinically, ACT-1004-1239 showed dose-dependent efficacy in various animal models such as of multiple sclerosis (Pouzol et al., 2021a) and acute lung injury (Pouzol et al., 2021b) using its ligands CXCL11 and CXCL12 as biomarker of target engagement. Dose-dependent increases in CXCL11 and CXCL12 plasma concentration were associated with a reduction in immune cell infiltrates into the central nervous system (Pouzol et al., 2021c) or the bronchoalveolar space (Pouzol et al., 2021b). ACT-1004-1239 is rapidly absorbed in rats. It displays a high clearance and a volume of distribution in excess of total body water (Richard-Bildstein et al., 2020). So far, two clinical studies with ACT-1004-1239 were conducted and showed favorable safety and tolerability profiles over the investigated dose range of 1–200 mg, following single- (Huynh et al., 2021b) and multiple-dose (Huynh et al., 2021a) administration in healthy humans. Pharmacokinetic (PK) and pharmacodynamic (PD) profiles suggested a once-daily dosing regimen in further clinical studies. In male and female subjects, ACT-1004-1239 was rapidly absorbed (time to reach maximum plasma concentration [t_max_] 1-3 h) and its exposure increased dose-dependently between 1 and 200 mg (Huynh et al., 2021a; Huynh et al., 2021b). At doses ≥10 mg, ACT-1004-1239 distributed to multiple compartments and was eliminated with a terminal half-life [t_1/2_] ranging from 18 to 24 h. Systemic exposure to ACT-1004-1239 reached steady-state conditions by Day 3 with almost no accumulation (Huynh et al., 2021a). Food had no relevant effect on the PK and the absolute bioavailability of ACT-1004-1239 was 53%. Furthermore, ACT-1004-1239 showed a large volume of distribution (183 L) and is considered a low-clearance drug in humans (Huynh et al., 2021b). Compared to male subjects, female subjects had overall higher ACT-1004-1239 exposure (Huynh et al., 2021a).

In the context of drug development, characterization of absorption, distribution, metabolism, and excretion (ADME) properties including but not limited to the identification of major metabolites is required to ascertain whether these may cause pharmacological or toxicological effects. Additionally, it provides key information on relevant drug interactions or dosing of special patient populations such as renally or hepatically impaired patients. Given the importance of understanding those characteristics, human ADME studies should be conducted during early clinical development (Spracklin et al., 2020). This can be achieved, for example, with a human microtracer component incorporated into a first-in human (FIH) study. The conduct of such integrated studies requires a highly sensitive analytical method for the determination of very low amounts of administered radioactivity by using accelerator mass spectrometry (AMS). AMS allows for detection of drug in the femtogram range, or below, by measuring the isotopic ratio of carbons (i.e., ^12^C/^14^C) (Graham and Garner, 2003; Lappin et al., 2006). Due to its high analytical sensitivity, AMS has a wide range of applications (Arjomand, 2010). In the context of early clinical drug development, AMS has been applied to assess various drug characteristics including but not limited to PK, mass balance, absolute bioavailability, and metabolite profiling using a microdose/microtracer approach (Boddy et al., 2007; Arjomand, 2010; Muehlan et al., 2018; Huynh et al., 2021b).

Here we report the metabolites and the metabolic pathways of ACT-1004-1239 in humans using results from *in vitro* and *in vivo* studies: 1) metabolite profiling in human liver microsomes (HLM) and identification of CYPs involved in the formation of the main metabolites, and 2) the ADME characteristics in humans investigated in the FIH study applying a microtracer approach (Huynh et al., 2021b). Metabolite structure elucidation of major human metabolites was supported by the use of rat samples. Data on the distribution of ACT-1004-1239 have been previously reported (Huynh et al., 2021b), and are therefore not covered herein.

## Materials and Methods

### Chemicals and Reagents

^14^C-ACT-1004-1239 was synthesized at Pharmaron (Cardiff, United Kingdom) in a stock solution containing 3% (v/v) 2 M hydrochloric acid in ethanol with a radioactive concentration of 1 mCi/ml and a specific activity of 58 mCi/mmol. The ^14^C-ACT-1004-1239 working solution used for *in vitro* studies was prepared by diluting an aliquot of the stock solution in a 1:1 (v/v) mixture of acetonitrile and water to reach a final concentration of 1 mM. This working solution was stored at –20°C. Prior to the experiments, the compound purity of the working solution was monitored. ^14^C-ACT-1004-1239 microtracer solution for oral administration in humans was prepared at the clinical site (Pharmaron, Baltimore, MD, United States). The ^14^C-ACT-1004-1239 stock solution was diluted to a final target microtracer concentration of 0.067 μCi/ml in a 5% (w/v) solution of mannitol and sterile water. The matching placebo oral solution consisted of 5% (w/v) mannitol and sterile water. ACT-1004-1239 capsules for clinical use were manufactured at Idorsia Pharmaceuticals Ltd. (Allschwil, Switzerland).

ACT-1004-1239 and synthetic reference standards of metabolites (M1, M23, and M38) were synthesized at Idorsia Pharmaceuticals Ltd. (Allschwil, Switzerland). For the NADPH-regenerating system, glucose-6-phosphate (disodium salt) and NADP^+^ were purchased from Sigma-Aldrich (Buchs, Switzerland) and glucose-6-phosphate dehydrogenase was supplied by Roche Diagnostics (Mannheim, Germany). Pooled HLMs were obtained from Becton Dickinson (Basel, Switzerland), while cDNA-expressed human CYPs (CYP1A1, CYP1A2, CYP2B6, CYP2C8, CYP2C9, CYP2C19, CYP2D6, CYP3A4, and control bactosomes) and co-expressing CYP reductase derived from *Escherichia coli* were purchased from Cypex Ltd. (Dundee, United Kingdom). The CYP-specific inhibitors furafylline, ketoconazole, quinidine, sulfaphenazole, and ticlopidine were purchased from Sigma-Aldrich (Buchs, Switzerland). Montelukast and N-benzylnirvanol were obtained from LKT Laboratories (St. Paul, MN, United States) and Becton Dickinson (Basel, Switzerland), respectively. All other chemicals and solvents used for analytical measurements were obtained from commercial sources.

### *In Vitro* Studies

Identification of the human CYPs metabolizing ACT-1004-1239 was performed using two complementary approaches: 1) incubation with HLMs in the absence and presence of CYP chemical inhibitors, and 2) experiments with recombinant CYPs.

#### Incubation With HLMs and CYP-Specific Inhibitors

^14^C-ACT-1004-1239 was incubated with HLMs at a final concentration of 10 μM. For this purpose, an aliquot of the ^14^C-ACT-1004-1239 stock solution was added to 100 mM phosphate buffer (pH 7.4) containing 1 mg/ml of microsomal protein. The reaction was initiated by addition of the pre-warmed NADPH-regenerating system and the incubation continued for 30 min at 37°C. The NADPH-regenerating system was prepared as a 10-fold concentrated stock solution. It consisted of 11 mM NADP^+^, 100 mM glucose-6-phosphate, 50 mM magnesium chloride in 0.1 M phosphate buffer (pH 7.4), and glucose-6-phosphate dehydrogenase (20 IU/ml). The latter was added shortly before the use of the NADPH-regenerating system. In the experiments with the competitive inhibitors sulfaphenazole (3 μM, CYP2C9 inhibitor), quinidine (1 μM, CYP2D6 inhibitor), montelukast (3 μM, CYP2C8 inhibitor), N-benzylnirvanol (5 μM, CYP2C19 inhibitor), and ketoconazole (1 μM, CYP3A4 inhibitor), a 5 μl-aliquot of the inhibitor stock solution was added to the reaction mixture prior to the initiation of the reaction. In incubations with the mechanism-based inhibitors, furafylline (20 μM, CYP1A2 inhibitor) and ticlopidine (0.5 μM, CYP2B6 inhibitor), the HLMs were pre-incubated for 10 min at 37°C in the presence of these inhibitors and the NADPH-regenerating system. The reaction was initiated by the addition of ^14^C-ACT-1004-1239. The organic solvent concentration in all incubations was kept at 1% (v/v). The reactions were terminated by the addition of one volume equivalent acetonitrile. The samples were centrifuged (for 10 min at 20,800 g and 10°C), and high performance liquid chromatography (HPLC) analysis (as described in *Analytical Method for Metabolite Profiling*) was applied to the resulting supernatants.

#### Incubations With Recombinant Human CYPs

^14^C-ACT-1004-1239 was incubated for 60 min with 100 pmol/ml of the respective recombinant CYP (CYP1A1, CYP1A2, CYP2B6, CYP2C8, CYP2C9, CYP2C19, CYP2D6, or CYP3A4) in 100 mM phosphate buffer (pH 7.4) at a final ^14^C-ACT-1004-1239 concentration of 10 μM. The incubation was initiated by addition of the pre-warmed NADPH-regenerating system and terminated by the addition of one volume equivalent acetonitrile. The NADPH-regenerating system was prepared as described in *Incubation With HLMs and CYP-Specific Inhibitors*. The organic solvent concentration in all incubations was kept at 0.5% (v/v). Control incubations in the absence of the NADPH-regenerating system or CYP, as well as incubations with control bactosomes, were performed in parallel under otherwise identical conditions. The samples were centrifuged for 10 min at 20,800 g and 10°C to obtain the supernatant which was used for HPLC analysis (as described in *Analytical Method for Metabolite Profiling*). The resulting data were further used to determine their contribution to the metabolic clearance *in vitro* by applying the intersystem extrapolation scaling approach as described by Chen et al. (2011).

#### Analytical Method for Metabolite Profiling

The HPLC-system for the recording of ^14^C metabolic profiles consisted of two Shimadzu pumps LC30AD (Shimadzu, Reinach, Switzerland) equipped with a Shimadzu column oven CTO-20A and a Shimadzu autosampler model SIL30AC. ^14^C-radiochemical detection was performed by a Berthold radioflow detector LB513 with a 200 μl-liquid cell Z-200-6M and an LB5036 pump for supplementing liquid scintillation cocktail at 3 ml/min (Berthold AG, Regensdorf, Switzerland). Data acquisition for metabolic profiling was done using the RadioStar software package (version 5.0.12.5; Berthold AG, Regensdorf, Switzerland).

Chromatographic separation of ^14^C-ACT-1004-1239 and its metabolites, derived from *in vitro* samples, was achieved on a Phenomenex Luna Phenyl-Hexyl column (250 × 4.6 mm ID, 3 μm, Torrance, CA, United States) at 40°C with a flow of 1 ml/min. Mobile phases consisted of 20 mM ammonium bicarbonate, adjusted to pH 8.6 with ammonium hydroxide (phase A), and acetonitrile (phase B). Parent drug and metabolites were eluted with the following linear gradient program: equilibration at 3 min, 10% B; 3-45 min, 10-30% B; 45-65 min, 30-40% B; 65-85 min, 40-50% B; 85-95 min, 50-85% B; 95-100 min, 85% B; 100-101 min, 85-10% B; 101-110 min, 10% B. Using these chromatographic conditions, ^14^C-ACT-1004-1239 had an average retention time of 67 min. The variability in retention time did not exceed 0.3 min.

### Human Study

#### Study Design

Before study initiation, the study was approved by IntegReview Institutional Review Board (Austin, TX, United States) and performed in accordance with Good Clinical Practice and the Declaration of Helsinki.

The investigation of mass balance and ADME characteristics reported here was incorporated in the single-center, randomized, double-blind, placebo-controlled FIH study (ClinicalTrials.gov: NCT03869320) assessing safety, tolerability, PK, and PD of single-ascending, oral doses of ACT-1004-1239 (1, 3, 10, 30, 100, and 200 mg) as previously reported (Huynh et al., 2021b).

Mass balance and the ADME characteristics were investigated at the 100 mg dose level. At this dose level, six subjects assigned to ACT-1004-1239 received in addition to the non-radioactive treatment concomitantly 9.2 µg (i.e., 1 µCi) ^14^C-ACT-1004-1239 as an oral solution. Two placebo subjects received a matching placebo solution.

Following dosing, all subjects stayed at the study site for at least 7 days. Each subject was requested to remain at the clinical site for an extension period until all subjects receiving active treatment met at least one of the following discharge criteria: individual cumulative radioactivity recovery in urine and feces >85% or total combined daily radioactive excretion in urine and feces in two consecutive 24 h collection intervals <1% of the administered dose. The end-of-study visit was on Day 14.

#### Study Population

For the assessment of mass balance and ADME characteristics, six healthy male subjects aged between 18 and 55 years with a body mass index of 18.0–30.0 kg/m^2^ were enrolled. Informed consent was obtained from each subject prior to any procedure and eligibility was determined based on various safety parameters including but not limited to vital signs, electrocardiogram (ECG), clinical laboratory data, physical examination, medical history, and previous exposure to radiation.

#### Sample Management: Collection, Processing, and Storage

Blood samples for the determination of total ^14^C-radioactivity PK in plasma and for metabolic profiling were drawn at pre-dose, 0.5, 1, 1.5, 2, 3, 4, 6, 8, 10, 12, 14, 16, 24, 36, 48, 60, 72, 96, 120, and 144 h post-dose. Following centrifugation for 10 min at 1800 g, plasma was collected and aliquoted. Urine samples were collected during the following intervals: pre-dose (within 24 h prior to dosing), 0-8, 8-16, and 16-24 h post-dose, and thereafter over 24 h intervals until Day 7. Feces samples were collected at pre-dose (within 72 h prior to dosing), and thereafter at 24 h intervals until Day 7. In subjects, who had to stay for the extension period, blood, urine, and feces samples were collected over additional 24 h intervals up to 240 h post-dose. Urine and feces samples obtained within a collection interval were pooled and aliquoted. Prior to aliquoting, the pooled feces sample was homogenized with water (1:1 to 1:2, w/w). All samples were stored at –70°C prior analysis at Pharmaron ABS (Germantown, MD, United States).

#### Pharmacokinetic Evaluation

PK parameters of total ^14^C-radioactivity in plasma were obtained by non-compartmental analysis using Phoenix WinNonlin (version 8.0, Certara, Princeton, NJ, United States). Individual measured plasma concentrations were directly used to determine t_max_ and maximum plasma concentration (C_max_), whereas area under the plasma concentration-time curve from 0 to infinity (AUC_0-∞_) was calculated according to the linear trapezoidal rule. The terminal t_1/2_ of total ^14^C-radioactivity was calculated with the following formula: t_1/2_ = ln(2)/λ_z_, where λ_z_ represents the terminal elimination rate constant.

#### Mass Balance

The amount of ^14^C-drug-related material in plasma, urine, and homogenized feces samples was analyzed using AMS (NEC SSAMS-250, National Electrostatics Corp., Middleton, WI, United States). Just prior to analysis, frozen samples were thawed and mixed in a multi-tube vortex for 5 min at 2000 rpm. Pre-dose samples were also analyzed for subtraction of inherent ^14^C background levels. Before drying all samples under vacuum, sodium benzoate (Alfa Aesar, Tewksbury, MA, United States) used as carbon carrier was added to plasma and urine samples in order to reach approximately 1.7 mg carbon (i.e., minimum amount of carbon required for AMS analysis) in the final samples. Dried samples were added to a sample oxidizer (Model 307, Perkin Elmer, Waltham, MA, United States) and processed to graphite prior loading those samples into cathodes of the AMS. The AMS determined isotope ratios (i.e., ^12^C^14^C) of each sample. For this purpose, negative carbon ions were produced through a caesium ion beam that was directed to the graphite-containing cathode. The resulting ions were extracted and forwarded to an accelerator, which caused electron stripping resulting in positively charged carbon ions. These ions were thereafter mass and charge separated by a magnet and an electrostatic analyzer, respectively. The total numbers of ^12^C+, ^13^C+, and ^14^C+ ions were finally counted. Raw data were expressed as percent modern carbon (pMC) and converted into radioactivity data with 100 pMC corresponding to 13.56 disintegrations per minute (dpm)/g carbon. These radioactivity data were further transformed into ng equivalents (ng-eq) per ml (plasma, urine) or per g (feces) based on the specific activity of the tracer and the dose administered.

#### Sample Preparation for Metabolite Profiling

For each subject, plasma samples were pooled based on the Hamilton pooling scheme (Hamilton et al., 1981) with samples collected within 72 h. Thereafter, a single cross-subject plasma pool was prepared by using a constant proportion of each individual Hamilton pool. Proportional pooling was applied for urine and homogenized feces samples. Individual pools were prepared using 0.05 and 0.75% of each urinary and homogenized fecal void collected over 72 and 144 h, respectively. A single cross-subject pool for both excreta was prepared by taking a sample representing 10% of each individual pool to obtain a representative pool of at least 95% of the total excreted ^14^C across all subjects.

Pooled plasma and feces samples were further purified by liquid-liquid extraction prior to HPLC analysis. The pooled plasma sample (4,000 μl) was extracted with 1200 μl acetonitrile:methanol (80:20, v/v) (Thermo Fisher Scientific, Waltham, MA, United States). After mixing for 1 min, the sample was centrifuged for 10 min at 3,750 rpm and 10°C followed by a drying process at room temperature under a stream of nitrogen. Thereafter, the supernatant was collected, dried, and aliquoted for analysis. These extraction steps were repeated two additional times with the remaining pellet to obtain a high extraction recovery. The homogenized feces sample (200 mg) was extracted by addition of 800 μl acetonitrile, followed by centrifugation for 5 min at 3,750 rpm and 4°C. The supernatant was collected and dried with nitrogen at 40°C. The remaining pellet was extracted by addition of 800 μl acetonitrile:water (1:1, v/v) followed by a drying process with nitrogen. The dried extract was reconstituted with acetonitrile:mobile phase A (see *Analytical Method for Metabolite Profiling*) (1:9, v/v) and vortexed for 5 min. The extraction recoveries were 93.6 and 86.9% for plasma and feces, respectively. Urine samples were directly used for analysis.

#### Metabolite Profiling

Metabolite profiles were generated by using HPLC together with an AMS detector. The plasma extract (120 μl), the feces extract (10 μl), and urine (100 μl) were injected onto the HPLC (Agilent 1290, Agilent Technologies, Santa Clara, CA, United States) equipped with a binary pump G4220A (Agilent Technologies), an autosampler G4226A (Agilent Technologies), a column compartment G1316C (Agilent Technologies), and a fraction collector G1364C (Agilent Technologies). Chromatographic separation of ^14^C-ACT-1004-1239 and its metabolites was achieved at 40°C with a flow rate of 1 ml/min. Mobile phases consisted of 20 mM ammonium formate adjusted to pH 9.5 with ammonium hydroxide (phase A) and a 7:2:1 (v/v/v) mixture of acetonitrile, methanol, and mobile phase A (phase B). Parent drug and metabolites were eluted with the following linear gradient program: equilibration at 3 min, 10% B; 3-10 min, 10-25% B; 10-20 min, 25% B; 20-30 min, 25-30% B; 30-60 min, 30% B; 60-70 min, 30-35% B; 70-90 min, 35% B; 90-100 min, 35-40% B; 100-110 min, 40-70% B; 110-120 min, 70-95% B; 120-125 min, 95% B; 125-126 min, 95-10% B; and 126-135 min, 10% B. Using these chromatographic conditions, ^14^C-ACT-1004-1239 had an average retention time of 107 min. The variability in retention time did not exceed 0.8 min The reference standards were used to assess the reproducibility of the method and to confirm the retention times. Column recovery, which met the acceptance criteria (i.e., 80-120%), was investigated with AMS by measuring ^14^C concentration of each sample prior and after analysis by HPLC. Following HPLC analysis, pools of HPLC fractions were prepared by taking equivalent portions of consecutive individual fractions for analysis with AMS (SSAMS-250, National Electrostatics Corp., Middleton, WI, United States). The amounts of ^14^C in each fraction and fraction pool were summed and compared to the solution injected onto the HPLC for the determination of profile recovery.

### Metabolite Identification and Structure Elucidation *In Vivo*

#### Rat

Following the human ADME study, metabolite profiling data from rats were retrospectively compared to the ones of humans. Rat metabolite profiling data were obtained from bile-duct cannulated animals (*n* = 2) after intravenous administration of 1.7 mg/kg (200 μCi/kg) ^14^C-ACT-1004-1239. Rat urine samples were selected for metabolite identification as this matrix contained the major metabolites observed in humans (i.e., M1 and M23). Moreover, urine samples were preferred over plasma, bile, or feces samples due to their relatively large volumes and metabolite concentrations, that could be directly analyzed without extensive sample preparation.

Rat urine samples collected up to 72 h post dosing were centrifuged for 10 min at 20,800 g and aliquots of 25 µl were directly injected onto the liquid chromatography combined with high resolution mass spectrometry (LC-HRMS) system.

Chromatographic separation of ^14^C-ACT-1004-1239 and its metabolites derived from rat urine samples was achieved on a Phenomenex Gemini NX-C18 column (250 × 4.6 mm ID, 3 μm, Torrance, CA, United States) using otherwise the same analytical method as described in *Metabolite Profiling*. Using these chromatographic conditions, ^14^C-ACT-1004-1239 had an average retention time of 109 min. The variability in retention time did not exceed 0.5 min.

LC-HRMS analysis was performed on an LTQ Orbitrap Velos Pro (Thermo Scientific, San Jose, CA, United States) in positive and negative heated electrospray ionization mode with source voltage at 3.0 and 2.5 kV, respectively. A capillary temperature at 300°C and nitrogen sheath gas flow rate at 40 arbitrary units were used. The orbitrap resolution was set at 60,000 for full scan mode and 15,000 for MS^n^. Fragmentation experiments were performed with a collision energy set at 35% for MS^n^ using collision induced dissociation (CID) and high energy collision induced dissociation (HCD). Full scan LC-HRMS chromatograms were generated to identify metabolites using the Xcalibur 3.0 and Compound Discoverer 1.0 software packages (Thermo Electron, San Jose, CA, United States). Selected ion chromatograms of standard metabolic transformations were generated and checked for the presence of signals in the respective chromatograms. The presence of metabolites was confirmed by accurate mass measurement and structure elucidation was performed using MS^n^. Finally, metabolite identity was confirmed by comparing the retention time, calculated/exact mass, and MS fragment pattern with the corresponding data of reference compounds.

#### Human

Structures of metabolites that were not observed in rats were identified at A&M Labor für Analytik und Metabolismusforschung Service GmbH (Bergheim, Germany) with LC-HRMS. The LC-HRMS system consisted of an analytical column (Gemini NX-C18, 250 × 4.6 mm, 3 µm, Phenomenex Inc., Torrance, CA, United States), a guard column (C18, 4 × 3 mm, Phenomenex Inc.), a binary HPLC-pump Series 1290 (Agilent Technologies, Waldbronn, Germany), an HTC-PAL autosampler (CTC Analytics AG, Zwingen, Switzerland), column oven Series 1260 (Agilent Technologies), and a QExactive mass spectrometer (Thermo Scientific, Bremen, Germany). The samples were fractionated with HPLC using identical conditions as described in *Metabolite Profiling*. Due to the low amount of drug-related material excreted via the urinary tract and the low abundance of unknown metabolites within this matrix (see results *Cumulative Recovery of* ^*14*^ *C-Radioactivity (Mass Balance) in Urine and Feces*; *Metabolite Profiling in Human Plasma, Urine, and Feces*), only metabolites in pooled plasma and feces samples were identified. In order to compare LC-HRMS and AMS results, sample preparation and the chromatographic method at A&M were identical to those at Pharmaron ABS. For the mass spectrometric analysis, heated electrospray ionization was performed in both positive and negative ionization mode with a source voltage at 3.5 kV and −3.5 kV, respectively. A capillary temperature at 300°C and nitrogen sheath gas flow rate at 65 arbitrary units were used. The orbitrap mass resolution was set at 140,000 for full scan mode and 17,500 for MS^2^. Fragmentation experiments were performed with a collision energy set at 20, 35, 50, and 65% for MS^n^ using HCD. Mass chromatograms were acquired with the Xcalibur 3.0 and 4.2 and data evaluation was supported by Compound Discoverer 3.1 software package (Thermo Scientific).

In total, four synthetic references (ACT-1004-1239, M1, M23, and M38) were available to support metabolite identification of non-radioactive material. The most abundant metabolites seen in the AMS chromatograms and M38 were identified by comparing the retention time, calculated/exact mass, and the MS^2^ fragment pattern with the corresponding data of reference compounds. Structures of unknown metabolites (i.e., no synthetic reference available) were further elucidated by the interpretation of correlating fragment masses and fragment structures of non-radioactive material. Metabolites previously identified in preclinical studies were denoted with the letter “M,” whereas the ones not observed in rats and only identified during this clinical study were denoted with the letter “A”. The numbers following the letters were consecutively assigned based on the appearance of the metabolites within different stages of drug development.

## Results

### *In Vitro* Studies

#### Incubation With HLMs and CYP-Specific Inhibitors

**FIGURE 1:**
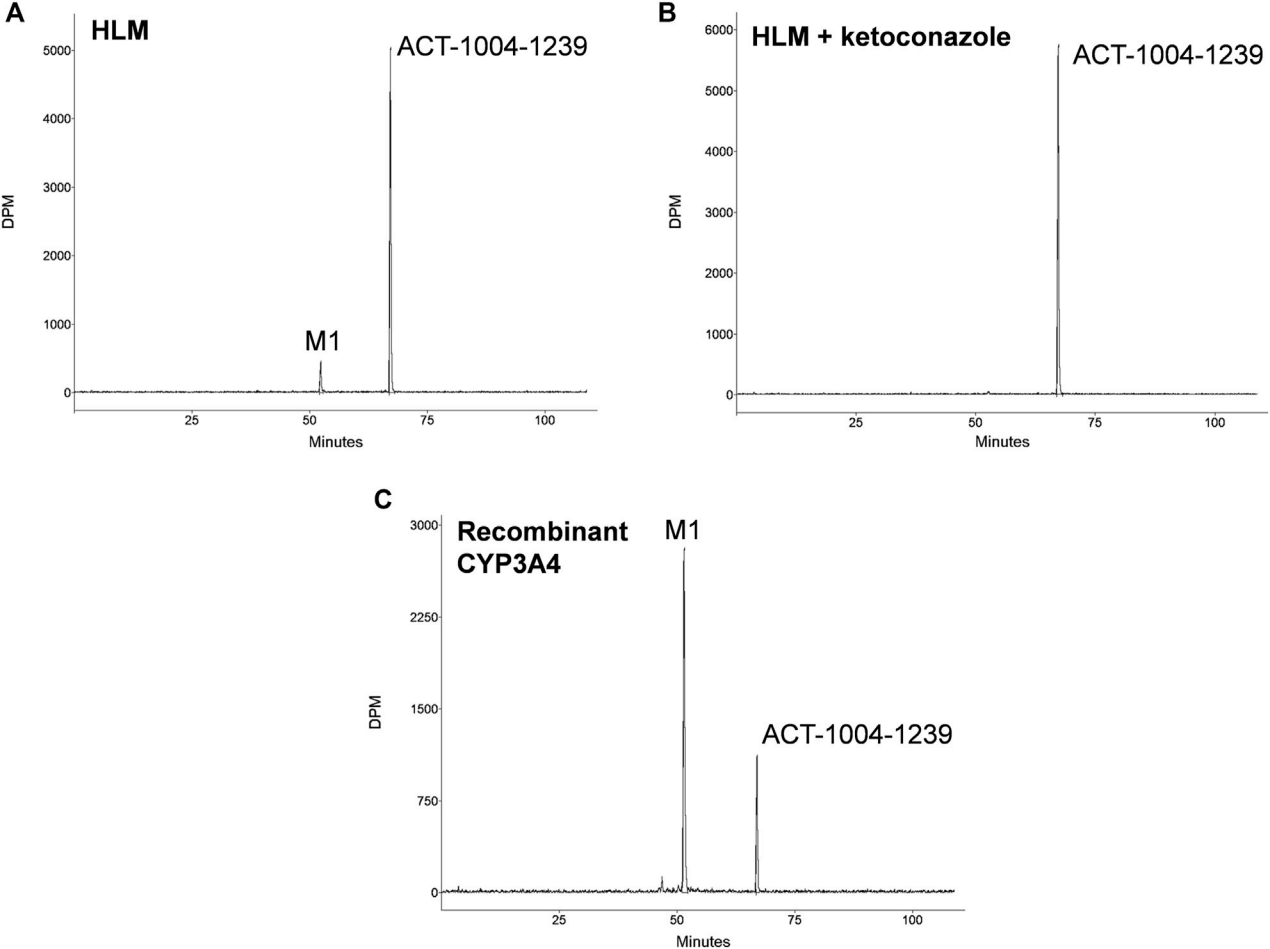
*In vitro* studies: metabolic profiles of ACT-1004-1239 following incubation with **(A)** human liver microsomes (HLM), **(B)** HLM and 1 μM ketoconazole, or **(C)** recombinant CYP3A4.

Incubations of ^14^C-ACT-1004-1239 with HLMs exclusively produced the metabolite M1 (Figure 1A). M1 was formed in the range of 7.5–12.0% of total chromatogram radioactivity. No metabolites were observed in the absence of the NADPH-regenerating system or HLMs. Testing the impact of validated inhibitors revealed ketoconazole as a potent inhibitor, where formation of M1 was completely abolished suggesting that CYP3A4 predominantly catalyzed this reaction (Figure 1B). In contrast, the presence of inhibitors of CYP1A2, CYP2B6, CYP2C8, CYP2C9, CYP2C19, and CYP2D6 had no effect on M1 formation (data not shown).

#### Incubation With Recombinant Human CYPs

Incubation of ^14^C-ACT-1004-1239 with recombinant CYPs (CYP1A1, CYP1A2, CYP2B6, CYP2C8, CYP2C9, CYP2C19, CYP2D6, and CYP3A4) showed that ACT-1004-1239 was metabolized by CYP1A1, CYP2C8, CYP2C19, and CYP3A4. M1 was, as in the experiments with HLMs, the only metabolite formed. A representative chromatogram of ACT-1004-1239 metabolism by recombinant CYPs is provided in Figure 1C. Based on the intersystem extrapolation scaling approach, CYP3A4 was the main CYP catalyzing the formation of M1 accounting for 94% of total M1 formation, and thus, total turnover *in vitro*. CYP2C19 and CYP2C8 contributed 5.2 and 1.2% to M1 formation, respectively. The contribution of CYP1A1 to M1 formation was not further investigated due the low liver abundance of CYP1A1 in human (Stiborová et al., 2005).

### Human Study.

#### Study Population

For the assessment of mass balance and ADME characteristics, six healthy male subjects received ^14^C-ACT-1004-1239 concomitantly with non-radioactive ACT-1004-1239. The mean (range) age and body mass index of these subjects were 31.2 (26.0-43.0) years and 25.4 (21.0-29.6) kg/m^2^, respectively. Most subjects were Black or African American (*n* = 5) and one subject was White. All subjects continued the extension period for additional collection of plasma, urine, and feces.

#### Pharmacokinetics of Total ^14^C-Radioactivity

**FIGURE 2:**
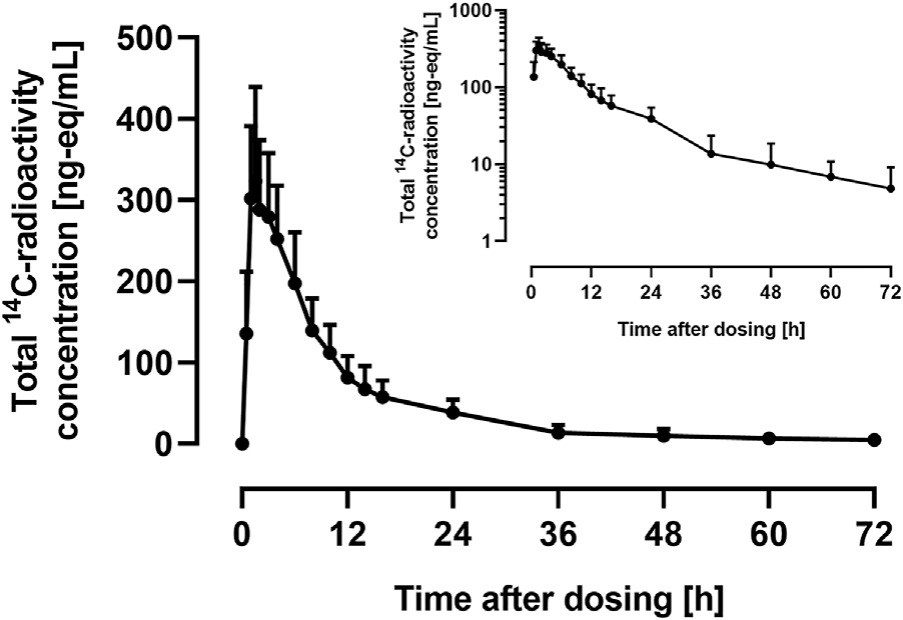
Arithmetic mean (+SD) concentration vs. time profile of total ^14^C-radioactivity in plasma on linear and semilog (inset) scale following a single oral administration of 9.2 μg ^14^C-ACT-1004-1239 on top of 100 mg non-radioactive ACT-1004-1239 (*N* = 6).

The arithmetic mean +SD plasma concentration of total ^14^C-radioactivity after administration of 9.2 μg ^14^C-ACT-1004-1239 and 100 mg ACT-1004-1239 is shown in Figure 2.

Following administration of ^14^C-ACT-1004-1239, drug-related material was rapidly absorbed. Disposition of ^14^C-drug-related material was covered by at least two compartments. After 72 h post-dose, the concentration of ^14^C-drug-related material was not measurable any longer (i.e., below the limit of quantification). PK parameters of total ^14^C-radioactivity in plasma are provided in Table 1.

**TABLE 1:** Pharmacokinetic parameters of total ^14^C-radioactivity in human plasma.

	t_max_ (h)	C_max_ (ng-eq/ml)	AUC_0-∞_ (ng-eq^*^h/ml)	t_1/2_ (h)
Geometric mean (95% CI)	1.5 (1.0-3.0)^a^	325 (240-439)	3,759 (2,738-5,162)	34.6 (16.8-71.4)
CV_ln_ (%)	-	29	31	78

a Data is displayed as median (range).

AUC_0-∞_, area under the plasma concentration-time curve from 0 to infinity; CI, confidence interval; C_max_, maximum plasma concentration; CV_ln_, geometric coefficient of variation; t_1/2_, terminal half-life; t_max_, time to reach maximum plasma concentration.

#### Cumulative Recovery of ^14^C-Radioactivity (Mass Balance) in Urine and Feces

**FIGURE 3:**
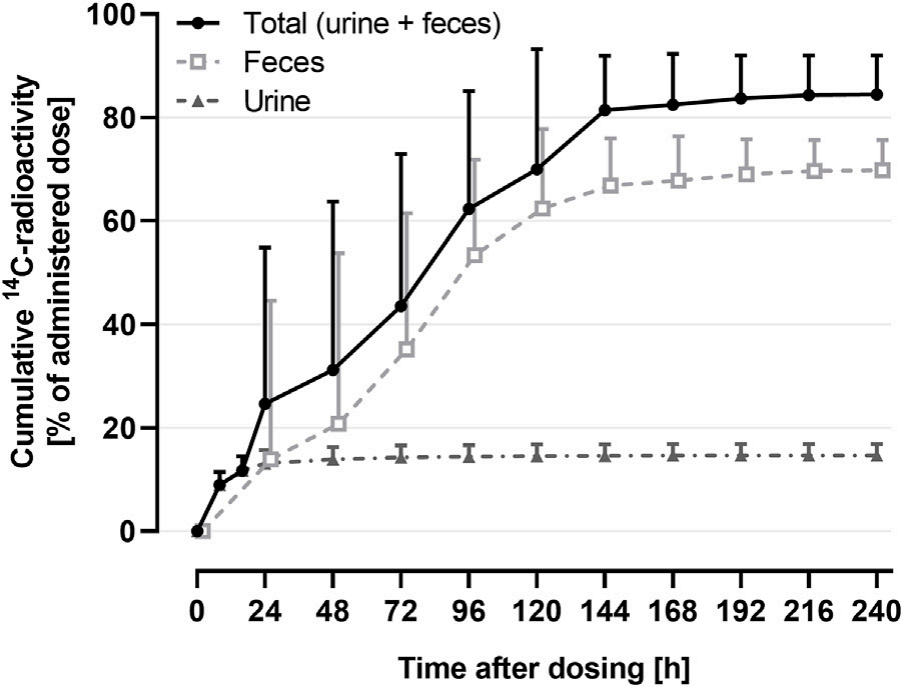
Arithmetic mean (+SD) cumulative recovery vs. time profile of total ^14^C-radioactivity in urine and feces (shown as % of administered dose) following a single oral administration of 9.2 μg ^14^C-ACT-1004-1239 on top of 100 mg non-radioactive ACT-1004-1239 (*N* = 6).

The mass balance using the cumulative recovery of ^14^C-drug-related material collected in urine and feces is shown in Figure 3.

The geometric mean (95% confidence interval) cumulative recovery of total radioactivity was 84.1% (76.8-92.2) of dose administered. ^14^C-drug-related material was mainly eliminated in feces with a mean cumulative recovery in feces and urine of 69.6% (63.8-75.9) and 14.5% (12.5-16.9), respectively.

#### Metabolite Profiling in Human Plasma, Urine, and Feces

**FIGURE 4:**
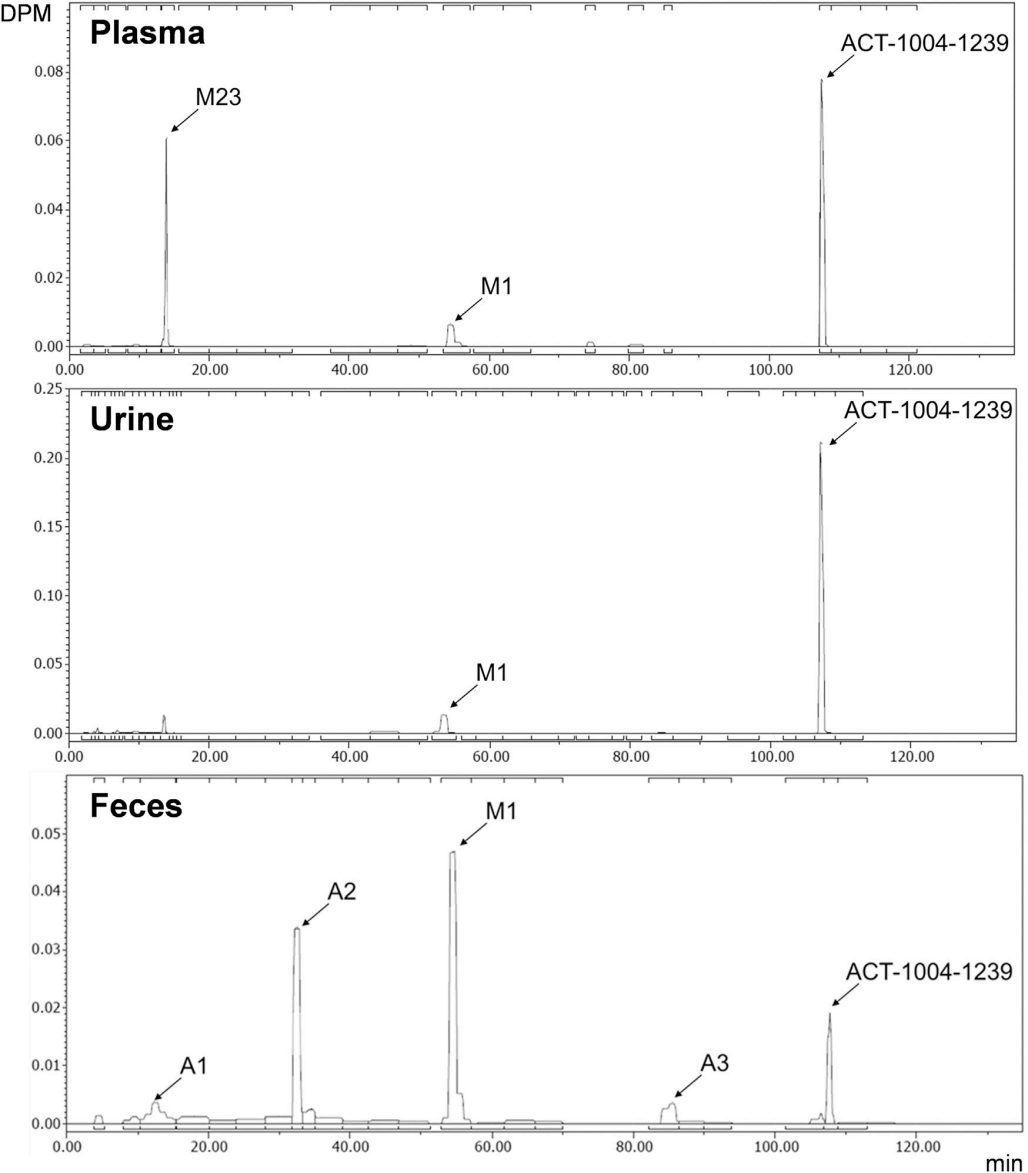
Metabolic profiles of ACT-1004-1239 in human plasma (top), urine (middle), and feces (bottom) pools. Squared brackets placed above and below each radiochromatogram indicate metabolites with a relative abundance of <5%.

Radiochromatograms of the human plasma and feces extracts, and urine sample were generated to identify ACT-1004-1239 and its radioactive metabolites. The radiochromatogram of each matrix is shown in Figure 4. The identities of ACT-1004-1239 and its metabolites, M1 and M23, were confirmed using reference compounds. Table 2 summarizes the relative abundancies of ACT-1004-1239 and its metabolites in plasma, urine, and feces samples as well as the percentages of ^14^C-radioactive dose administered in urine and feces.

**TABLE 2:** Summary of relative abundance of ACT-1004-1239 and its metabolites.

Compound ID	Plasma	Urine		Feces		Urine + feces
	% in sample	% in sample	% of dose^a^	% in sample	% of dose^a^	% of dose
ACT-1004-1239	53.9	69.6	10.1	7.5	5.2	15.3
M1	10.3	9.7	1.4	34.1	23.7	25.1
M23	21.1	-	-	-	-	-
A1	-	-	-	5.8	4.0	4.0
A2	-	-	-	22.0	15.3	15.3
A3	-	-	-	3.9	2.7	2.7
Other^b^	14.7	20.7	3.0	26.7	18.6	21.6
Total	-	-	14.5	-	69.6	84.1

a Calculations were based on geometric mean of total cumulative excretion in urine/feces.

b Refers to the sum of metabolites with a relative abundance of <5%.

In the plasma sample, unchanged ACT-1004-1239 was the most abundant entity identified with a relative abundance of 53.9% of total radioactivity. In addition, there were two major circulating metabolites (defined as >10% of total drug exposure [AUC]), i.e., M1 and M23 accounting for 10.3 and 21.1% of total radioactivity in plasma, respectively. All other metabolites accounted for <5% of total radioactivity and were therefore not identified.

In urine, the most dominant entity detected was unchanged ACT-1004-1239 with a relative abundance of 69.6% of total radioactivity of the urine sample. Besides this, M1 was observed with much lower abundance (9.7% of total radioactivity of the urine sample). All other metabolites were significantly lower in abundance (i.e., <5% of total radioactivity in the urine sample) and were therefore not identified.

In feces, there were two dominant entities observed, i.e., M1 and A2, which accounted for 34.1 and 22.0% of total radioactivity of the feces sample, respectively. Unchanged ACT-1004-1239 and the metabolites A1 and A3 were seen with a relative abundance of 7.5, 5.8, and 3.9% of total radioactivity of the feces sample, respectively. All other metabolites were not identified due to their low signal abundance (i.e., <5% of total radioactivity in the feces sample).

Based on the cumulative recovery of ^14^C-drug-related material, 25.1% of the administered ^14^C-radioactive dose was excreted as M1 (1.4% in urine, 23.7% in feces) and 15.3% each as unchanged ACT-1004-1239 (10.1% in urine, 5.2% in feces) as well as A2 (in feces only). All other metabolites were excreted with <5% of ^14^C-radioactive dose (Table 2).

### Metabolite Identification and Structure Elucidation *In Vivo*

#### Rat

**FIGURE 5:**
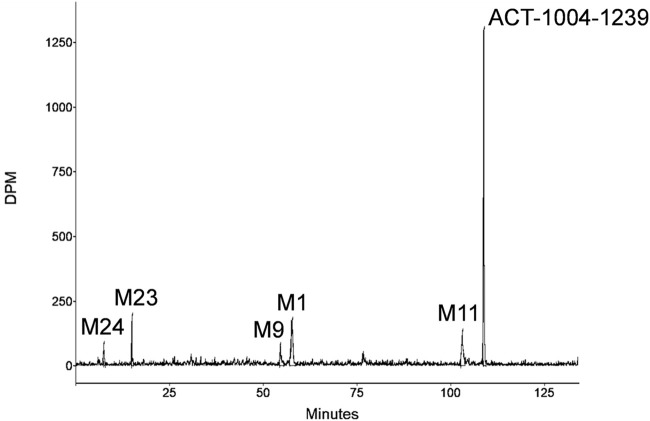
Representative metabolic profile of ACT-1004-1239 in rat urine after intravenous dosing of ^14^C-ACT-1004-1239.

**FIGURE 6:**
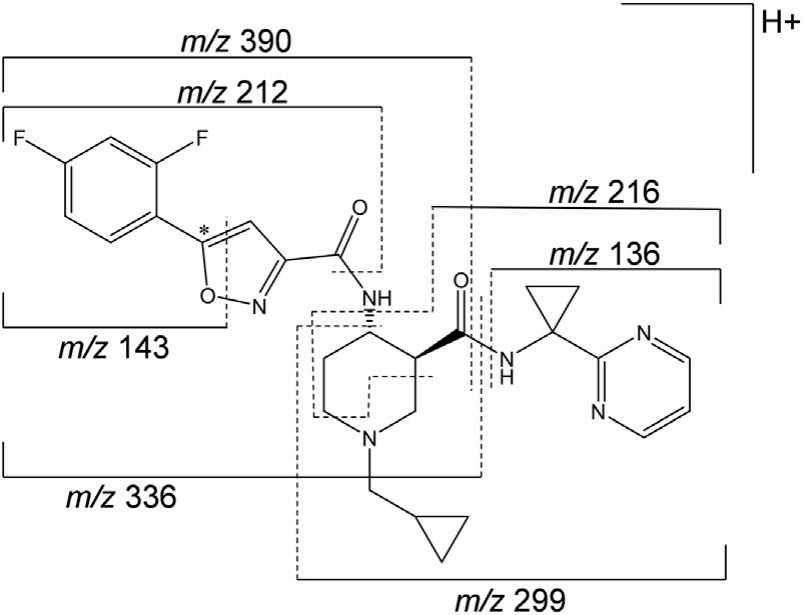
Mass spectrometric fragmentation pattern of ^14^C-ACT-1004-1239. The asterisk (*) indicates the position of the ^14^C label, which was taken into consideration for the applicable accurate fragment masses (*m/z*). The fragment ion with m/z 212 includes in addition to the depicted pattern two hydrogen atoms.

**FIGURE 7:**
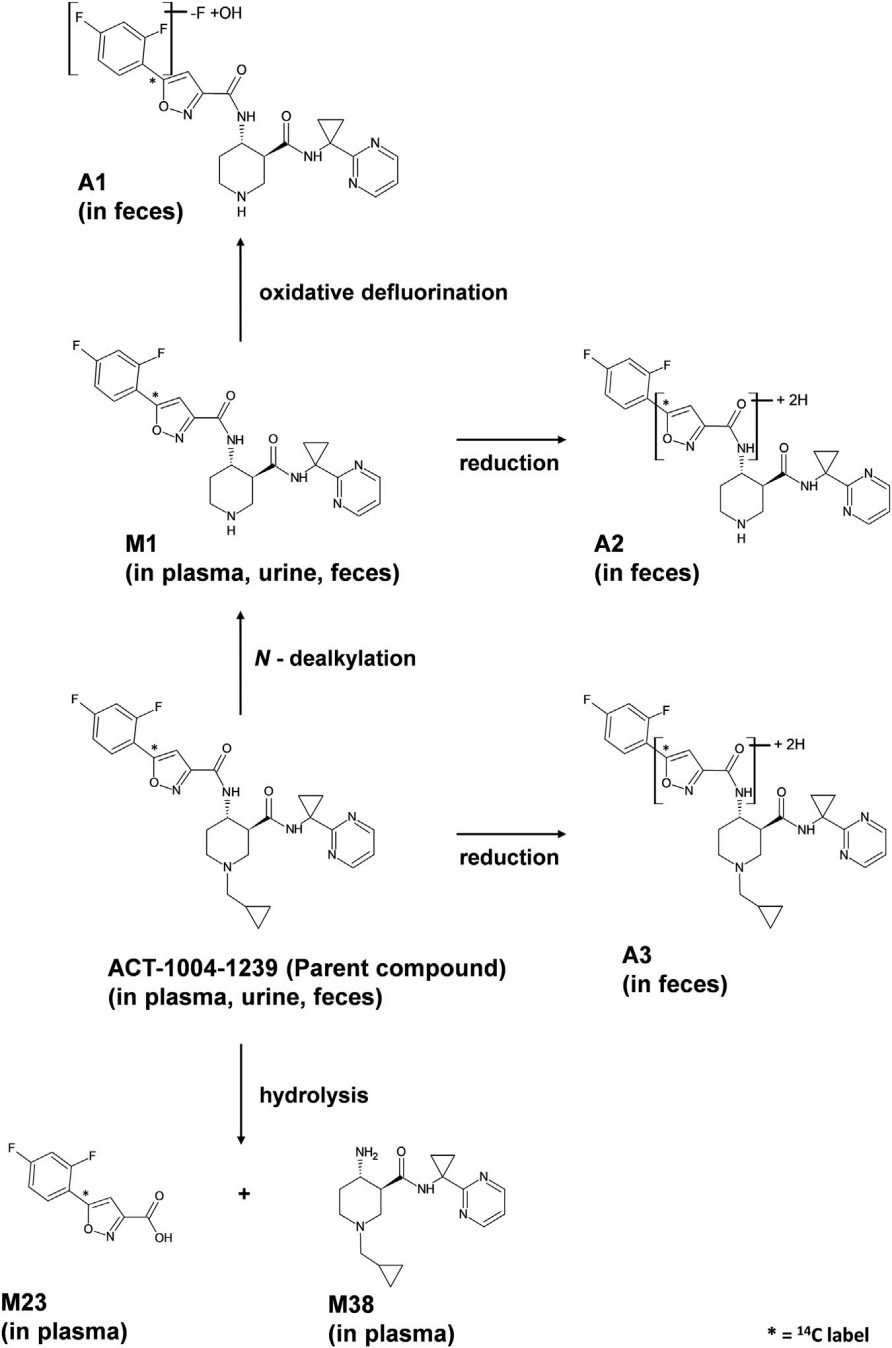
Proposed metabolic pathways of ACT-1004-1239 in humans.

A representative radiochromatogram of rat urine containing the major human metabolites (i.e., M1 and M23) is shown in Figure 5. Besides ^14^C-ACT-1004-1239, M1, and M23, also metabolites M9, M11, and M24 were detected. However, M9, M11, and M24 were not observed in humans with an abundance >5% per matrix (Figure 4), and therefore, their structure will not be further discussed. Based on the structural elucidation of M23, it was suggested that a counterpart metabolite M38 may exist which does not carry the radiolabel. This may explain the absence of M38 in the radiochromatogram of rat urine. To confirm presence of M1, M23, and M38 in urine, synthetic references were used for unequivocal structural identification. The mass spectrometric outputs (including, amongst others, accurate and calculated/exact masses, and *m/z* values of diagnostic fragment ions) of ^14^C-ACT-1004-1239, M1, M23, and M38 from rat urine are provided in Table 3. The accurate mass of each fragment provided in the MS spectra was compared to the calculated/exact mass. The MS key fragmentation pattern of ^14^C-ACT-1004-1239 (m/z 525) is shown in Figure 6. Structures of ^14^C-ACT-1004-1239, M1, M23, and M38 are shown in Figure 7, and were elucidated and identified in rat urine as described below.

•^14^C-AC T-1004-1239
  The fragmentation patterns of ^14^C-ACT-1004-1239 ([M + H]^+^ *m/z* 525.2292) did not differ significantly between the ion trap CID or HCD fragmentation techniques. Therefore, all fragment ions reported herein refer to either of the two techniques. Additional insight into structural elucidation was achieved using MS^3^ fragmentation of the most abundant MS^2^ fragment ions. In positive ionization mode, the most intense fragment ion was with *m/z* 390. Less abundant fragment ions were fragments with *m/z* 336, 299, 216, and 136.
•M1
  With a [M + H]^+^ *m/z* 471.1823 M1 was identified as a secondary amine metabolite resulting from the loss of the methyl cyclopropyl moiety (i.e., oxidative N-dealkylation) of the parent. This was consistent with the absence of fragment ions with *m/z* 390 and 299, and the presence of the fragment with *m/z* 336 with two other fragments with *m/z* 216 and 136. The structure was confirmed using its chemical reference as shown in the supplemental material (Supplementary Figure S1).
•M23
  M23 was identified as the difluorophenyl isoxazole carboxylic acid metabolite resulting from the central amide bond hydrolysis of the parent. This metabolite was not detected in positive ionization mode but in negative ionization mode. In negative ionization mode, the theoretical exact mass of [M-H]^-^ *m/z* 226 was not detected. However, an ion with [M-H]^-^ *m/z* 182.0302 was seen instead, most likely following loss of CO_2_ in the source. MS^2^ fragmentation of this ion yielded two fragments, i.e., with *m/z* 162 following loss of hydrogen fluoride and with *m/z* 138 corresponding to the loss of ^14^CCH_2_O. The structure was confirmed using its chemical reference as shown in the supplemental material (Supplementary Figure S2).
•M38
  M38, with a [M + H]^+^ *m/z* 316.2132 was identified as the counterpart of M23 resulting from the central amide bond hydrolysis of the parent. This was consistent with the presence of fragment ions with *m/z* 299 and 216. The structure was confirmed using its chemical reference as shown in the supplemental material (Supplementary Figure S3).

**TABLE 3:** Mass spectrometric identification of ^14^C-ACT-1004-1239 and its metabolites in rat urine.

Positive ionization mode					
Compound	Retention time (min)	Accurate mass [M + H]^+^	Calculated/exact mass [M + H]^+^	Diagnostic fragment ions (MS^n^)	Identity
ACT-1004-1239	106.5	525.2292	525.2296	390, 336, 299, 216, 136	**-**
M1	54.4	471.1823	471.1827	336, 216, 136	- C_4_H_6_ (oxidative N-dealkylation)
M38	19.9	316.2132	316.2132	299, 216	- C_10_H_3_F_2_NO_2_ (hydrolysis)
**Negative ionization mode**					
**Compound**	**Retention time (min)**	**Accurate mass [M-H]^-^**	**Calculated/exact mass [M-H]^-^**	**Diagnostic fragment ions (MS^n^)**	**Identity**
M23^a^	13.4	182.0302	182.0299	182, 162, 138	- C_17_H_23_N_5_O+ O (hydrolysis)

For all compounds, except M38, the presented data refer to radiolabeled material (e.g., ^14^C-ACT-1004-1239).

a Source fragmentation, the [M-H]- ion was not directly observed, [M-CO_2_-H]- was detected instead.

#### Human

Metabolites that were not identified in rat urine but were observed with considerable abundance in human feces (Figure 4) were A1, A2, and A3. In order to elucidate the structures of these unknown metabolites, their calculated/exact mass and MS^2^ fragment patterns were compared with the ones of ACT-1004-1239 and M1. The mass spectrometric outputs (including amongst others accurate and calculated/exact masses and *m/z* values of diagnostic fragment ions) of ACT-1004-1239, M1, A1, A2, and A3 from human feces are provided in Table 4. The MS key fragmentation pattern of ^14^C-ACT-1004-1239 (m/z 525) is shown in Figure 6. The structures of these unknown metabolites were elucidated and identified in human feces as described below.

•A1
  A1 was identified as an analog of M1 after oxidative defluorination at the difluorophenyl moiety (fragment ion with *m/z* 141). This was consistent with the MS^2^ fragmentation spectra, which showed a fragment ion with *m/z* 139 representing a hydroxyfluorobenzoic acid fragment instead of *m/z* 141 and a fragment ion with *m/z* 332 instead of *m/z* 334. Other fragment ions (with *m/z* 136 and 216) were available in both spectra. However, it remains unknown which of the two fluorine atoms was substituted with a hydroxy function. The MS2 spectra of A1 and M1 [M + H]^+^ ions are provided in the supplemental material (Supplementary Figure S4).
•A2
  A2 was identified as a reduced analog of M1 after addition of two hydrogen atoms. The fragment ions with *m/z* 136, 141, and 216 were present in the spectra of A2 and M1, whereas the fragment ions with *m/z* 210 and 336 were only detectable in the MS^2^ spectrum of A2. This indicated that the addition of two hydrogen atoms had taken place at the isoxazole moiety of the molecule which may have led to ring opening resulting in an imine and hydroxy group. The MS2 spectra of A2 and M1 [M + H]^+^ ions are provided in the supplemental material (Supplementary Figure S5).
•A3
  A3 was identified as a reduced analog of the parent compound after addition of two hydrogen atoms. Upon comparison of MS^2^ spectra of ACT-1004-1239 and A3, the fragment ions with *m/z* 136, 141, and 216 were present in both spectra, whereas the fragment ions with *m/z* 210 and 390 were only detectable in the spectrum of A3. All other diagnostic fragment ions were similar to the fragments of ACT-1004-1239. This indicated that the addition of two hydrogen atoms had taken place at the isoxazole moiety of the molecule which may have led to ring opening resulting in an imine and hydroxy group. The MS2 spectra of A3 and parent drug [M + H]^+^ ions are provided in the supplemental material (Supplementary Figure S6).

**TABLE 4:** Mass spectrometric identification of ACT-1004-1239 and its metabolites in human feces.

Compound	Retention time (min)	Accurate mass [M + H]^+^	Calculated/exact mass [M + H]^+^	Diagnostic fragment ions (MS^n^)	Identity
ACT-1004-1239	107.6	523.2265	523.2264	388, 334, 216, 141, 136	-
M1	55.7	469.1795	469.1794	334, 216, 141, 136	- C_4_H_6_ (oxidative N-dealkylation)
A1	12.0	467.1851	467.1838	332, 216, 139, 136	M1 - F + OH (oxidative defluorination)
A2	33.0	471.1949	471.1951	336, 216, 210, 141,136	M1 + H_2_ (reduction)
A3	85.6	525.2425	525.2420	390, 216, 210, 141, 136	+ H_2_ (reduction)

Data are displayed in positive ionization mode.

For all compounds, the presented data refer to non-radiolabeled material (e.g., ACT-1004-1239).

Taken together, based on the results from metabolite profiling, identification, and elucidation, the metabolic pathways in humans are proposed as depicted in Figure 7. The elimination of ACT-1004-1239 via M1 was the only metabolic pathway that contributed to ≥25% of elimination:

• Oxidative N-dealkylation of ACT-1004-1239 to M1 (representing 25.1% of ^14^C-radioactive dose). M1 thereafter undergoes two notable reactions: a) oxidative defluorination to A1 (representing 4.0% of ^14^C-radioactive dose) and b) reduction to A2 (representing 15.3% of ^14^C-radioactive dose).

Apart from this, a minor elimination pathway was via A3 (i.e., 2.7% of ^14^C-radioactive dose).

## Discussion

ACT-1004-1239 is an orally available, potent, and selective first-in-class CXCR7 antagonist that showed efficacy in preclinical animal models of multiple sclerosis (Pouzol et al., 2021a) and acute lung injury (Pouzol et al., 2021b), and had a favorable clinical profile following single- (Huynh et al., 2021b) and multiple-dose (Huynh et al., 2021a) administration. In context of drug development, identification of metabolites and metabolic pathways of a drug is of utmost relevance. Therefore, *in vitro* and *in vivo* studies were conducted to 1) identify human enzymes involved in the metabolism of ACT-1004-1239 and 2) characterize ADME in humans with the use of rat samples for metabolite structure elucidation of major human metabolites.

*In vitro*, two complementary approaches (incubation with HLMs in the absence/presence of CYP-specific chemical inhibitors and experiments with recombinant CYPs) were chosen to identify the CYPs involved in the metabolism of ACT-1004-1239. It was shown that ACT-1004-1239 was particularly metabolized to M1 via phase I biotransformation enzymes including CYP1A1, CYP2C8, CYP2C19, and CYP3A4. Additional analyses using the intersystem extrapolation scaling approach (Chen et al., 2011) revealed that CYP3A4 was the main contributor to ACT-1004-1239 metabolic clearance, accounting for 94% of total turnover *in vitro*.

The ADME characteristics in humans were investigated in the context of an FIH study applying a microtracer approach (Huynh et al., 2021b). Such integrative studies have previously been successfully conducted (Muehlan et al., 2018, Muehlan et al., 2019). The integration of a human ADME study in early clinical studies (e.g., FIH study) is an innovative approach to accelerate clinical development (Muehlan et al., 2018). It enables characterization of metabolites during the early stage of clinical development, which is encouraged by United States Food and Drug Administration (FDA) (United States Department of Health and Human Services Food and Drug Administration Center for Drug Evaluation and Research, 2020b) and it allows to enhance resource efficiency. Indeed, for the integrated approach only very low amounts of radiolabeled compound are needed, so that no determination of radiation burden is required, and hence, no quantitative whole-body autoradiography in rodents is to be conducted. In addition, the very low amounts used do not require to be produced according to Good Manufacturing Practice (Ufer et al., 2017; Spracklin et al., 2020). However, for such an integrative approach a highly sensitive technique is needed, which is realized by the application of AMS (Graham and Garner, 2003; Lappin et al., 2006). In contrast to most conventional ADME studies, the conduct of such integrative studies typically uses different formulations for non-radioactive and radioactive drug. This could possibly result in non-homogenous absorption resulting in unequal quantitative data between non-radioactive and radioactive material. Retrospective quantitative analysis of non-radioactive parent drug and metabolites using the standard addition approach confirmed their relative abundance in plasma as observed for radioactive equivalents provided in Table 1. These data support the homogeneity in absorption in this study.

Based on the results obtained in the human ADME study, it was shown that the concentration-time profile of total ^14^C-radioactivity was similar to the one of non-radioactive ACT-1004-1239 (Huynh et al., 2021b) with comparable t_max_ and C_max_. However, the geometric means of AUC_0-∞_ and terminal t_1/2_ of total ^14^C-radioactivity were approximately 2-fold higher than the ones of 100 mg non-radioactive ACT-1004-1239. This suggests that the metabolism of ACT-1004-1239 in humans results in metabolites, which have a lower elimination rate, and thus, cause the increase in AUC_0-∞_ and t_1/2_ as previously observed (Roffel et al., 2017; Muehlan et al., 2019). Following oral administration of 9.2 μg ^14^C-ACT-1004-1239, the mean cumulative recovery of radioactivity in urine and feces was approximately 84%, which is above the generally acceptable threshold for human ADME studies (>80%) (Roffey et al., 2007). These data are in line with previous observations showing that compounds with a radioactivity t_1/2_ < 50 h achieve at least 80% of cumulative recovery (Roffey et al., 2007). Investigation of mass balance revealed that ACT-1004-1239 was predominantly eliminated *via* feces and to a lesser extent in urine. Based on the totality of radioactivity recovered in feces and urine, it is unlikely that ACT-1004-1239 is eliminated *via* other elimination routes. This is supported by the physicochemical properties of ACT-1004-1239 (Richard-Bildstein et al., 2020).

Profiling the human plasma samples for metabolites revealed two major circulating metabolites (i.e., M1 and M23) besides the parent compound. As these metabolites were also present in rats (i.e., not human-specific), their structures were elucidated using highly concentrated rat urine samples. M1 is the product of oxidative N-dealkylation with loss of the cyclopropylmethyl moiety from the parent. On the other hand, M23 results most likely from hydrolysis of the central amide bond of the parent. Exploratory *in vitro* studies investigating enzymes that could catalyze the formation of M23 excluded the involvement of carboxylesterases (i.e., CES1b, CES1c, and CES2) which are known to hydrolyze amide bonds (Idorsia Pharmaceuticals Ltd., data on file). Additional investigations with other hydrolases such as amidases or epoxide hydrolases are to be considered in order to understand formation of M23 (Magdalou et al., 2003). Formation and structural elucidation of M23 suggested the theoretical presence of its metabolite counterpart. This metabolite was detected and identified as M38. As it was devoid of the radiolabel, M38 was not detectable in the rat radiochromatogram. However, the analysis using MS confirmed the presence of M38 in rat urine. Similar to rats, M38 was not detectable in the human radiochromatograms. Nevertheless, quantification of M38 in the pooled human plasma using a synthesized reference indicated that its presence was negligible, and hence, it is not considered a major circulating metabolite. In contrast to M1 and M38, M23 was not detectable in the positive ionization mode, but only in negative ionization mode, most likely as the weakly basic isoxazole ring did not allow for an efficient protonation (Palmer, 2003).

Major circulating metabolites constituted >10% of total drug exposure (AUC) like M1 and M23 have to be cautiously investigated for any relevant human safety concern (e.g., disproportionality). For this purpose, it is required to examine according to the guidance for *Safety Testing of Drug Metabolites* (United States Department of Health and Human Services Food and Drug Administration Center for Drug Evaluation and Research, 2020b) whether the human plasma exposure to such metabolites is distinctly greater than the maximum exposure in animals used for toxicological evaluation of ACT-1004-1239. This additional investigation is to be performed before the initiation of any large-scale clinical trials. Besides the identification of major circulating metabolites, another objective of a human ADME study is the identification of main metabolic pathways. For this purpose, the metabolites A1, A2, and A3 were elucidated in human feces. A1 was identified as a secondary product following oxidative defluorination of M1. This type of biotransformation reaction can be catalyzed by CYPs (e.g., CYP3A4 and CYP1A1/2) and/or oxygenases such as flavin-containing monooxygenases and was previously observed in the metabolism of other drugs (Xie et al., 2013; Amaya et al., 2018). The reductive metabolites, A2 and A3, were formed following reductive cleavage of the isoxazole ring (i.e., N-O bond) to an imine intermediate which was thereafter hydrolyzed. This ring opening may be explained by the chemical properties of the isoxazole ring containing, adjacent to the nitrogen, an oxygen atom with greater electronegativity which may increase the susceptibility to reduction (Dalvie et al., 2002). The reductive cleavage of the isoxazole ring was also observed with other xenobiotics and was catalyzed by the gut microflora, CYPs, and aldehyde oxidase (Mannens et al., 1993; Sugihara et al., 1996; Kalgutkar et al., 2003; Zhang et al., 2008). Additional exploratory investigations with, for example, gut microflora could aid in elaboration of the formation of A2 and A3. Except for A1, A2, and A3, no metabolites were structurally elucidated in human excreta, due to their significantly lower abundance (i.e., <5% of total radioactivity per excreta sample). M1 was the only known metabolite that was confirmed in human urine and feces. Taking the retention time and its appearance in rat urine into consideration, it is likely that M23 is also present in human urine. However, the identity of M23 in human urine sample was not further investigated and was therefore not confirmed.

Based on metabolite profiling results, only one metabolic pathway contributed to ≥25% of ACT-1004-1239 elimination:

• Oxidative N-dealkylation of ACT-1004-1239 to M1. M1 undergoes thereafter two reactions: a) oxidative defluorination to A1 and b) reduction to A2.

Since the reaction from ACT-1004-1239 to M1 is predominantly catalyzed by CYP3A4, it is recommended to clinically investigate possible drug-drug interactions of ACT-1004-1239 using strong index inhibitors and/or inducers of this enzyme (United States Department of Health and Human Services Food and Drug Administration Center for Drug Evaluation and Research, 2020a). In addition, according to health authorities guidelines (United States Department of Health and Human Services Food and Drug Administration Center for Drug Evaluation and Research, 2003; European Medicines Agency Evaluation of Medicines for Human Use, 2018), it is recommended to conduct PK studies in subjects with hepatic impairment when the hepatic metabolism and/or excretion accounts for a substantial portion (>20%) of the elimination of parent compound. Thus, such aforementioned PK studies should be conducted in the clinical development program of ACT-1004-1239.

## Conclusion

Taken together, integration of an ADME study using the microtracer approach in conjunction with the AMS methodology in an FIH study facilitated the elucidation of the metabolism of ACT-1004-1239, a first-in-class CXCR7 antagonist, in early clinical development. Results from *in vitro* and *in vivo* studies underpin the relevance of preclinical investigations to support the identification of metabolites in humans. In this human study, the disposition and metabolic pathways of ACT-1004-1239 were sufficiently characterized, allowing to determine potential successive clinical studies. It was shown that elimination of ACT-1004-1239 mainly occurred through feces following major metabolism via CYP3A4. Besides ACT-1004-1239, two major circulating metabolites were identified in human plasma.

## Data Availability

The original contributions presented in the study are included in the article/Supplementary Material, further inquiries can be directed to the corresponding author.
