# Supplementary material for: Absorption, Metabolism, and Excretion of ACT-1004-1239, a First-In-Class CXCR7 Antagonist: In Vitro, Preclinical, and Clinical Data
Source: Front Pharmacol. 2022 Mar 30;13:812065. doi: 10.3389/fphar.2022.812065 (PMC9006992; doi:10.3389/fphar.2022.812065)
Supplement: Supplementary file 1 [file DataSheet1.docx]

Supplementary Material


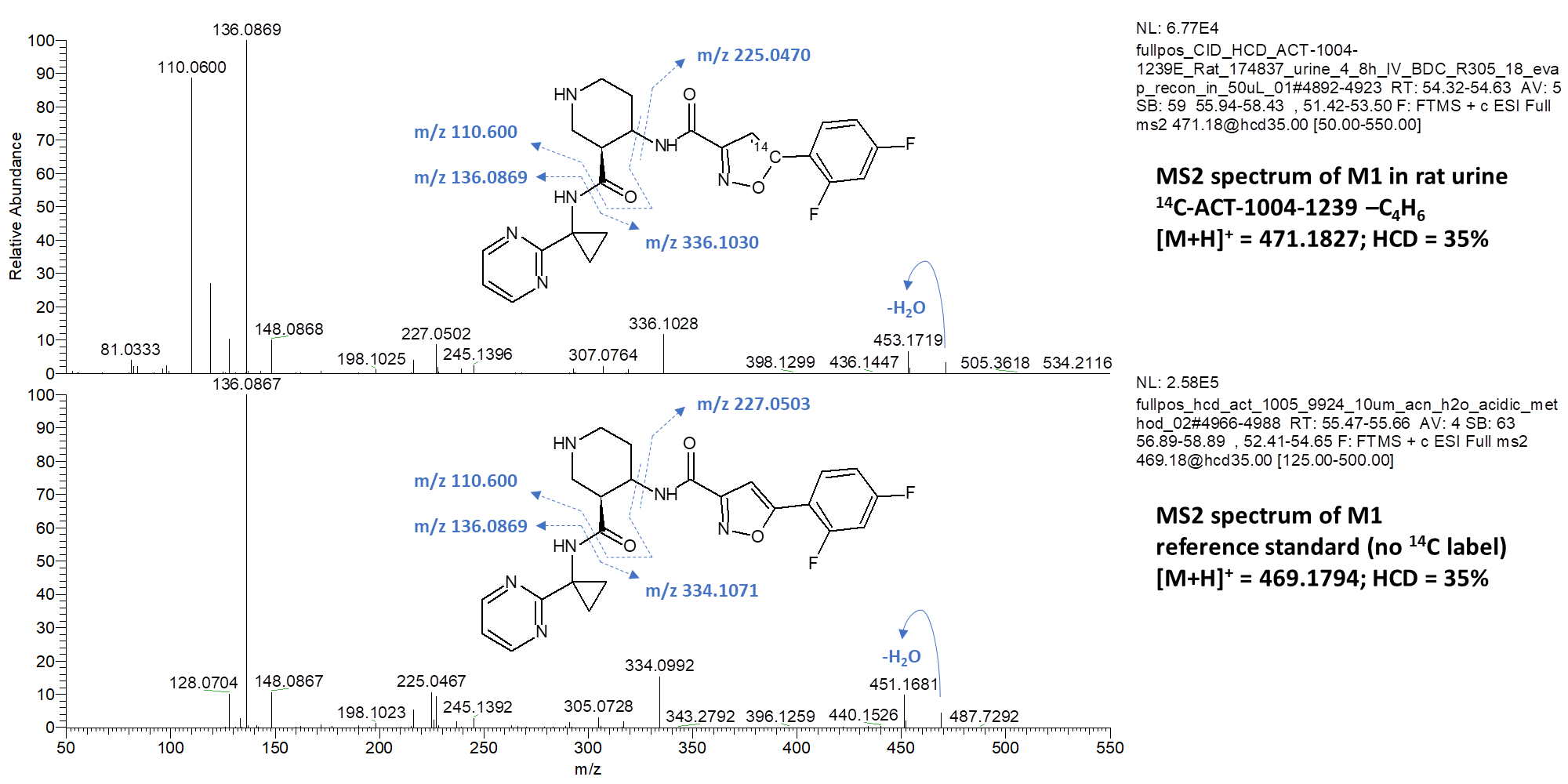


**Fig. S1**. **Structure elucidation of M1 in rat urine.** Figure shows the comparison of MS2 spectra of [M+H]^+^ ions of M1 (above) and reference standard (below) after a high energy collision-induced dissociation (HCD) of 35%. Spectrum differences in the range below m/z 125 are explained by different scan ranges applied for in vivo samples and references compound.


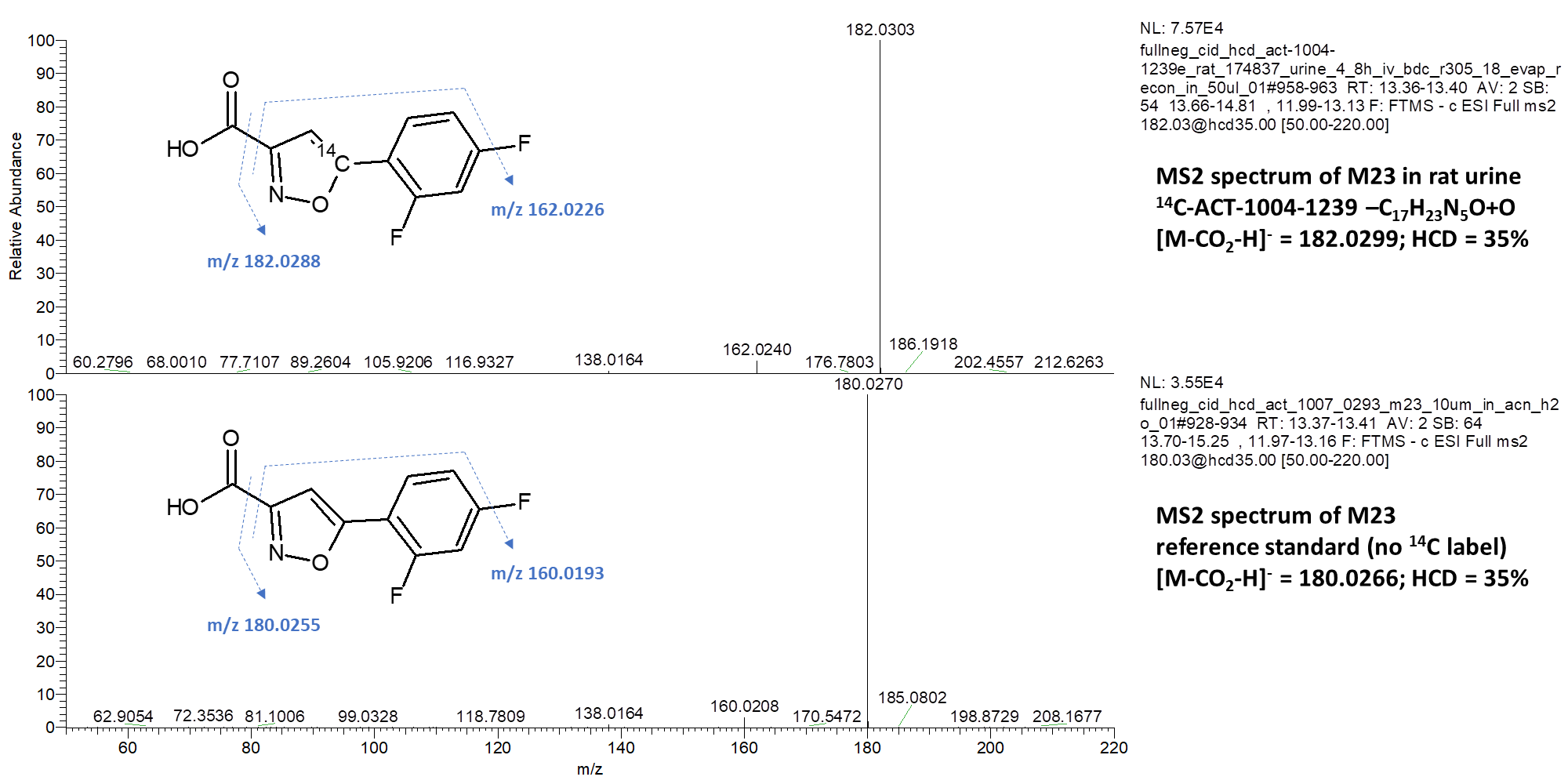


**Fig. S2**. **Structure elucidation of M23 in rat urine.** Figure shows the comparison of MS2 spectra of [M-CO_2_-H]^-^ ions of M23 (above) and reference standard (below) after a high energy collision-induced dissociation (HCD) of 35%.


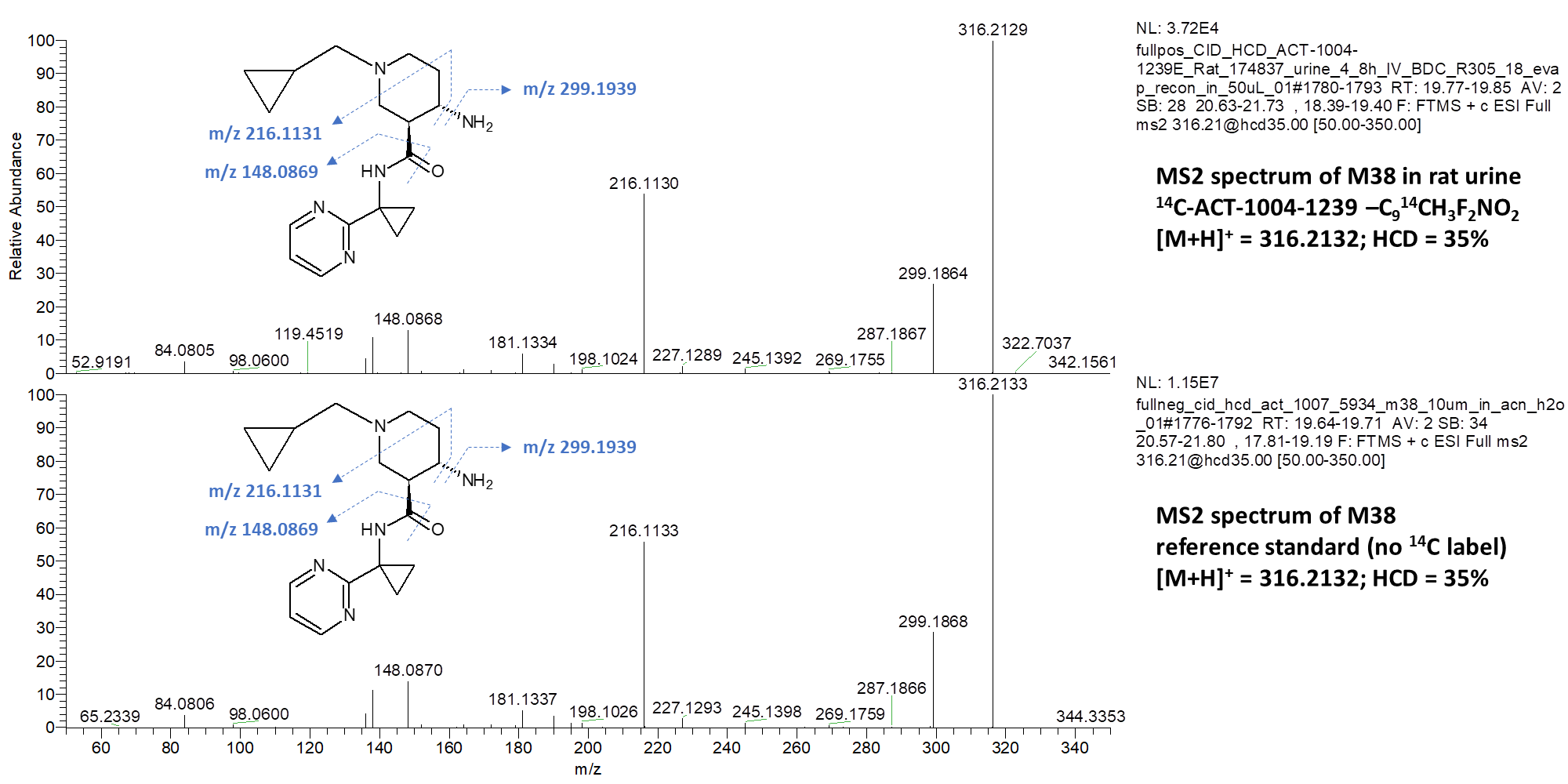


**Fig. S3**. **Structure elucidation of M38 in rat urine.** Figure shows the comparison of MS2 spectra of [M+H]^+^ ions of M38 (above) and reference standard (below) after a high energy collision-induced dissociation (HCD) of 35%.

**
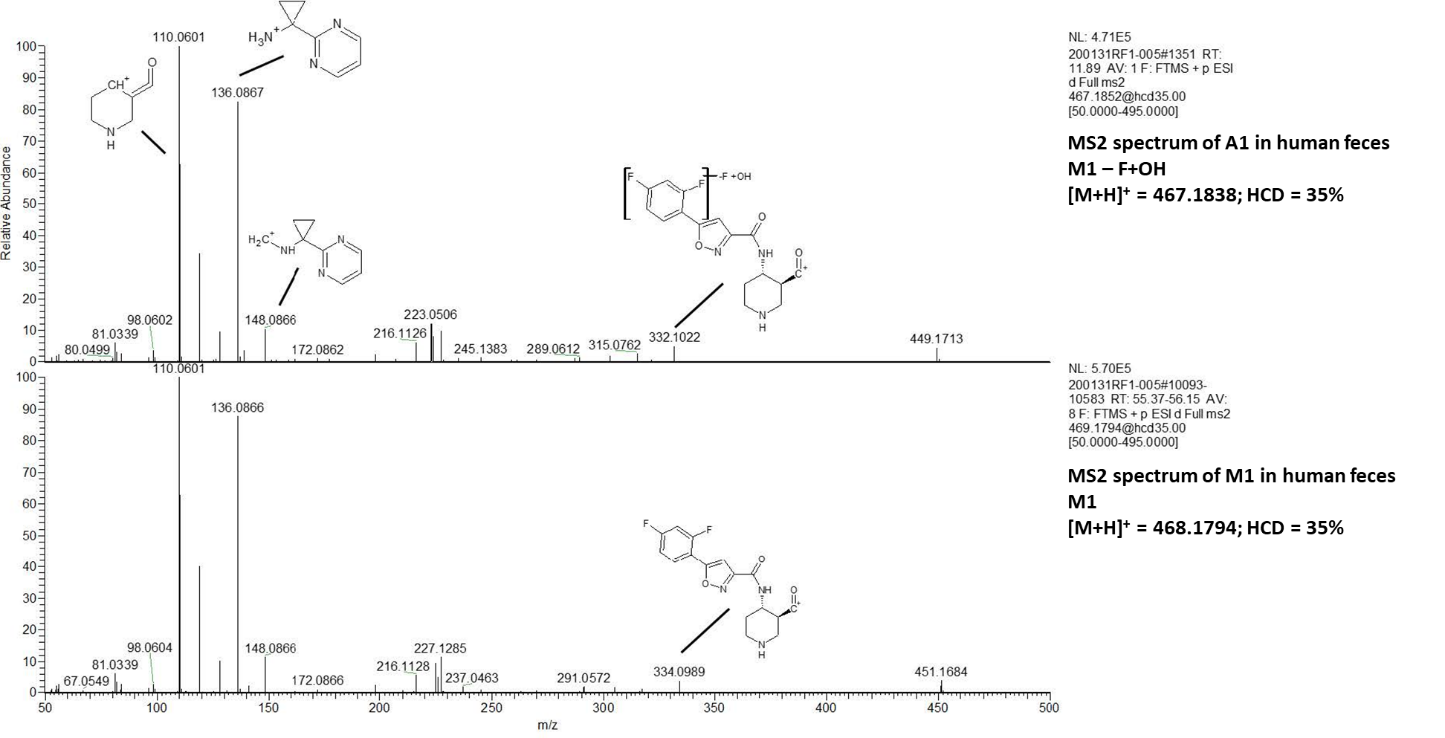
**

**Fig. S4**. **Structure elucidation of A1 in human feces.** Figure shows comparison of MS2 spectra of [M+H]^+^ ions of A1 (above) and M1 (below) after high energy collision-induced dissociation (HCD) of 35%.


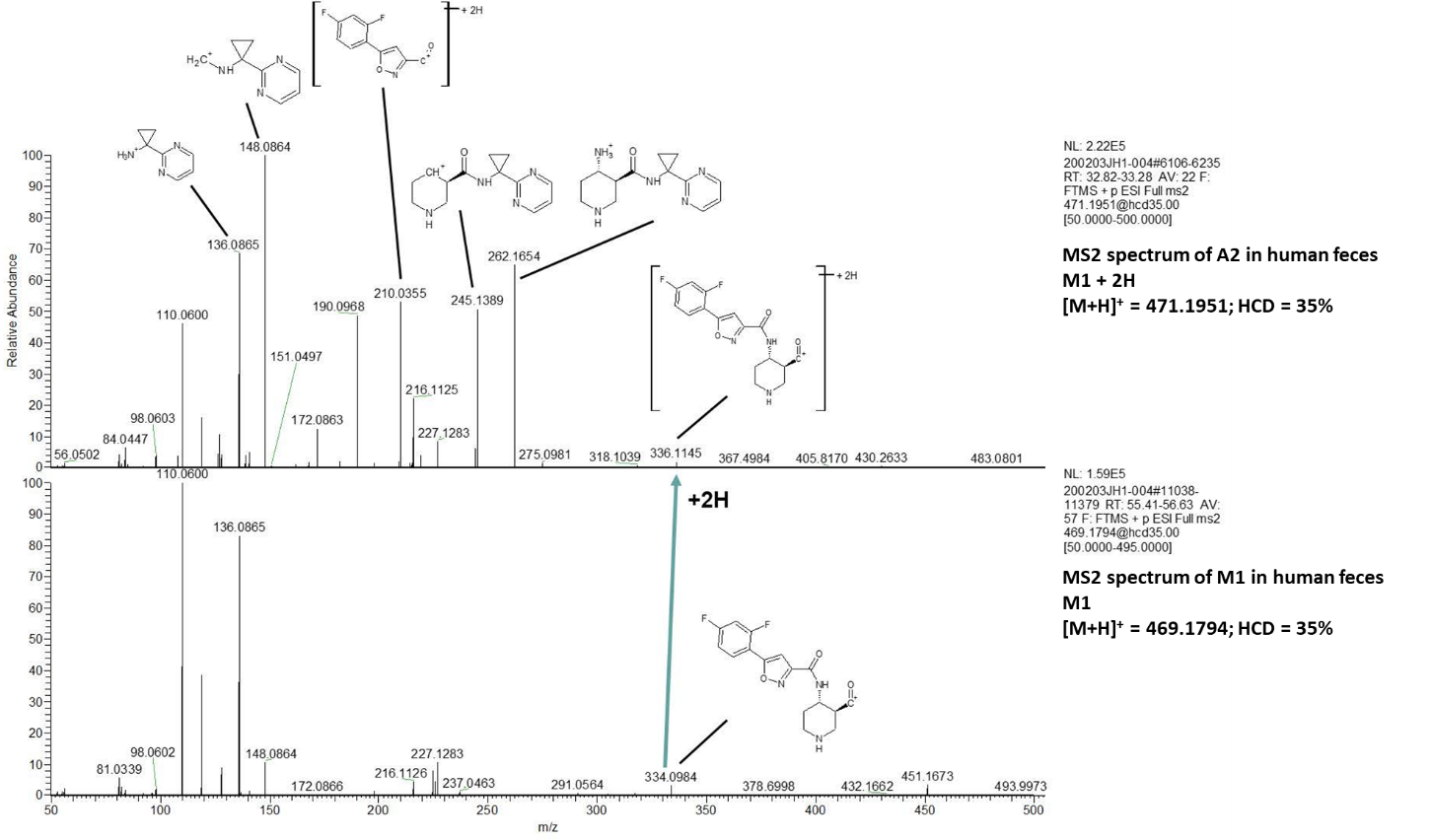


**Fig. S5**. **Structure elucidation of A2 in human feces.** Figure shows comparison of MS2 spectra of [M+H]^+^ ions of A2 (above) and M1 (below) after high energy collision-induced dissociation (HCD) of 35%.


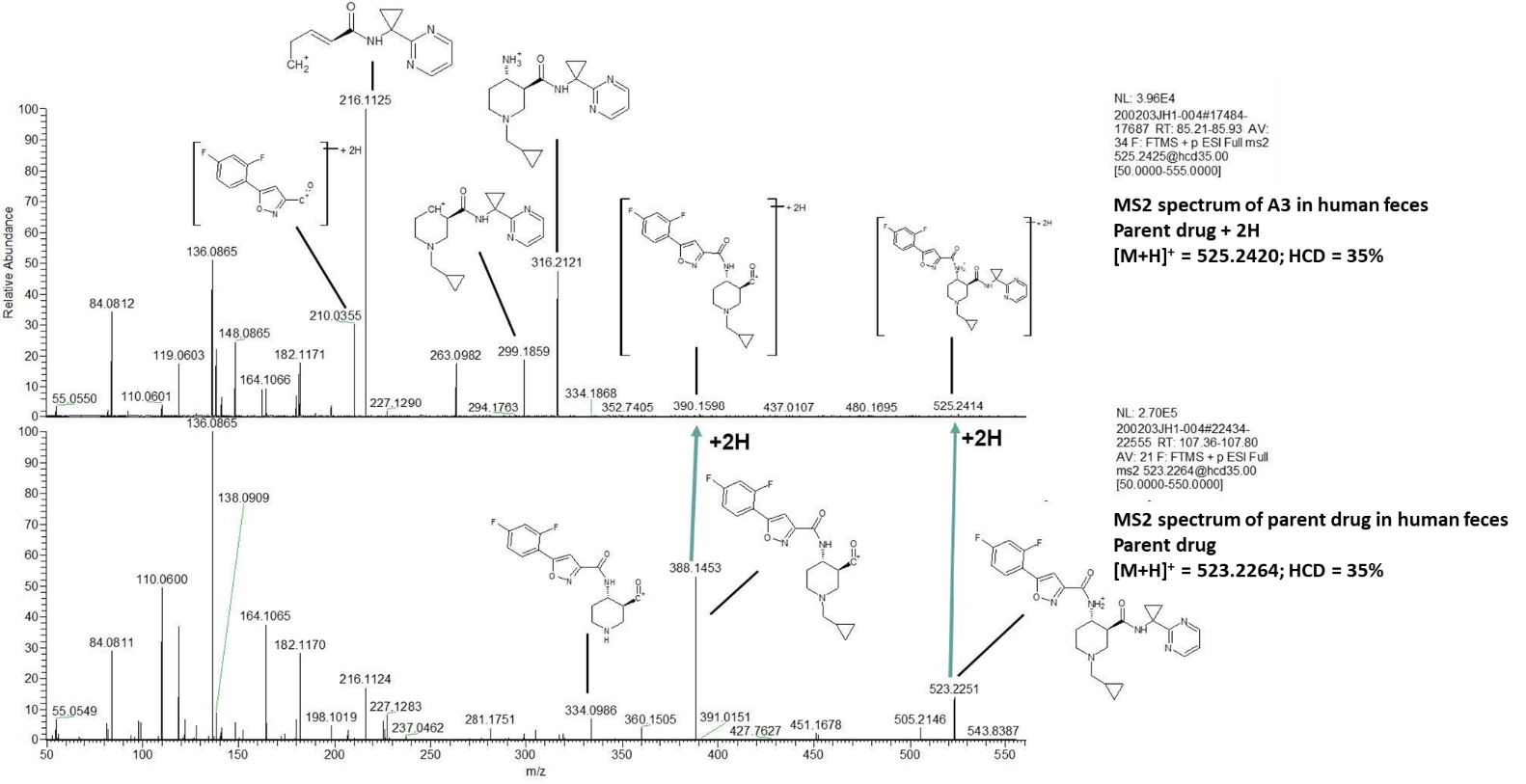


**Fig. S6**. **Structure elucidation of A3 in human feces.** Figure shows comparison of MS2 spectra of [M+H]^+^ ions of A3 (above) and parent drug (below) after high energy collision-induced dissociation (HCD) of 35%.
